# Comparison of resilience and quality of life between adolescent blood cancer survivors and those with congenital heart disease: a cross sectional study. 

**DOI:** 10.1186/s12955-020-01487-w

**Published:** 2020-07-14

**Authors:** Sunhee Lee, Nack-Gyun Chung, Jae Young Choi

**Affiliations:** 1 College of Nursing, The Catholic University of Korea, 222 Banpo-daero, Seocho-gu, Seoul, 06591 South Korea; 2 Department of Pediatrics, Seoul St. Mary’s Hospital, College of Medicine, The Catholic University of Korea, Seoul, South Korea; 3 Division of Pediatric Cardiology, Severance Cardiovascular Hospital, Yonsei University Health System, Seoul, South Korea

**Keywords:** Cancer, Chronic illness, Resilience, Quality of life

## Abstract

**Background:**

The resilience and Quality of Life (QOL) of adolescent cancer survivors was compared with those of children with other diseases to identify the patterns and factors that affect resilience and QOL The purpose of the present study was to compare the resilience and QOL between adolescent blood cancer survivors and adolescents with Congenital Heart Disease (CHD).

**Methods:**

A cross-sectional study was conducted in two hospitals. Ninety-four adolescent blood cancer survivors and 81 adolescents with CHD completed a self-reported questionnaire regarding resilience, QOL, and general characteristics. Independent t-test and ANCOVA were used to compare the resilience and QOL between adolescent blood cancer survivors and adolescents with CHD.

**Results:**

The resilience of adolescent blood cancer survivors was significantly lower than that of adolescents with CHD, and the QOL of adolescent blood cancer survivors was not different from that of adolescents with CHD.

**Conclusions:**

The experiences of adolescent blood cancer survivors were different from those of adolescents with CHD even though they are of the same ages. Adolescents with chronic disease have a different level of illness controllability and self-regulation according to their disease and situation. Therefore, health-providers need to develop the specific programs for improving resilience and QOL of adolescents with chronic illness with focusing their characteristics and situations.

## Background

Blood cancer is the most common cancer type among children and adolescents aged 0–14 years in the United State and Korea [1, 2]. Blood cancer is the second most common cause of death for children aged 0–14 years among various types of cancers in the United State and Korea [1, 3]. However, due to advanced treatment, the five-year survival rate of blood cancer among children aged 0–14 years is 85% in the United State as well as in Korea [1, 2]. Therefore, paediatric blood cancer was recently recognized as a chronic illness.

The majority of paediatric blood cancer survivors live to be adults, but they experience anxiety about recurrence and experience a high burden of late effects [4]. They also need to accomplish tasks according to developmental stage, which could cause psychological health problems. Because paediatric cancer survivors tend to have overall psychological difficulties compared to the general population [5], health providers should consider psychosocial functioning for these patients.

Quality of Life (QOL) may be considered to be one of the indicators of psychosocial outcome and resilience may influence paediatric cancer survivors’ QOL [6]. Many researchers used QOL and resilience as variables to investigate psychosocial functioning of adolescents with chronic illness [4, 5, 7, 8]. Resilience refers to the process of effectively negotiating, adapting to, or managing a significant source of stress or trauma [9]. Assets and resources within an individual and her/his life and environment facilitate the capacity for adaptation and ‘bouncing back’ in the face of adversity [9]. The adolescent resilience model (ARM) is designed to describe the processes and outcomes of resilience experienced by adolescents with cancer and other chronic illness [10]. The ARM consists of 3 classes of protective factors (individual, family, and social) and 2 classes of risk factors (individual and illness-related) [10]. These protective and risk factors affect the adolescent’s outcome of resilience and QOL [11]. Resilience plays a positive role as protective factors in response to stress and QOL refers to a global sense of well-being in the context of the ARM [11]. The resilience and QOL of patients with chronic disease can vary according to the disease, because patient adversity and process of adaptation to a chronic disease are heterogeneous [12]. For example, Limber et al. [13] explained that paediatric liver transplantation recipients reported lower QOL than children with type 1 diabetes. Accordingly, the resilience and QOL of paediatric cancer survivors in comparison to those of children with other diseases need to be determined in order to identify the patterns and factors that affect resilience and QOL.

Many researchers have studied the psychosocial issues of people with chronic illness. The researchers reported that the people who have strong internal health locus of control (the belief that they can control their health condition) was likely to learn self-management [14] and showed high QOL [15], self-esteem, and life satisfaction [16]. The people having cancer showed lower scores of internal health locus of control than those of healthy people [17] because they continuously experienced a fear of recurrence [18]. However the sure way to prevent cancer recurrence is not yet known. On the other hand, the scores of internal health locus of control in people with heart disease were higher than those in healthy people at comparing the two studies using the same scale for investigating internal health locus of control [17, 19]. These mean that psychological problems can be different in the people with cancer and heart disease, even though both diseases were considered as chronic disease. In addition, the leading causes of death for teenager population were accident, homicide, suicide, cancer and heart disease from 1999 to 2006 [20]. And the leading causes of death among patients aged one to 19 years were heart disease and cancer in 2014 [3]. Therefore, it is valuable to compare the resilience and QOL of adolescents blood cancer survivors, the resilience and QOL of adolescents with Congenital Heart Disease (CHD) was investigated. The purpose of the present study was to compare the resilience and QOL between adolescent cancer survivors and adolescents with CHD because there have not been sufficient comparative studies to date. Scientific investigation about the different points from the present study can be a cornerstone to develop the specific program for improving psychosocial functioning of adolescent cancer survivors. It was hypothesized that adolescent cancer survivors would show low levels of QOL and resilience compared to adolescents with CHD.

## Methods

### Study design

The cross-sectional study was conducted to investigate and compare resilience and QOL between adolescent blood cancer survivors and adolescents with CHD.

### Setting and participants

Researchers obtained the approvals of two university-affiliated tertiary hospitals which are located in the same city to secure a number of subjects. Each hospital has expertise in paediatric leukemia and CHD. To recruit adolescents blood cancer survivors, a research assistant visited an out-patient paediatric cancer clinic at a university-affiliated hospital, and a nurse practitioner recommended patients 12 to 20 years old, diagnosed with adolescents blood cancer, and under maintenance therapy or follow up after finishing treatment. Adolescent who can lead a daily life were included and adolescents currently suffering from complications were exluded. To recruit adolescents with CHD, a research assistant visited an out-patient paediatric cardiology clinic at another university-affiliated hospital, and a nurse practitioner recommended patients 12 to 20 years in age and diagnosed with CHD. Participants were recruited during summer break, from July to August, 2017, to maximize the amount of data. Initially, 106 adolescent cancer survivors and 81 adolescents with CHD took part in this study, but 12 adolescent cancer survivors failed to complete the questionnaire. In the end, 94 adolescent blood cancer survivors and 81 adolescents with CHD participated in this study. At a minimum sample size of 64 per group with a t-test for two groups, effect size was 0.5, and power was 0.80 from G*power 3.0.10 [21].

### Data collection

This study was approved by the Institute Review of Board of the two involved hospitals before data collection. Informed consent was obtained from participants over 18 years in age and from both participants and parents from patients under 18 years of age. Research assistants explained the aims and necessity of this study to the participants and parents, and the recruited adolescents who decided to take part in this study completed the questionnaire in the waiting room.

### Measurements

#### Resilience

The resilience scale developed by Wagnild and Young [22] was used to evaluate and compare the resilience between adolescent blood cancer survivors and adolescents with CHD. The Resilience Scale consists of two subscales: personal competence and acceptance of self and life. The possible range was from 25 to 175, and a higher score indicates higher resilience. Wagnild [23] reported that a score over 145 means high resilience and a score under 125 means low resilience. This self-reported instrument consisted of 25 items rated on a 7-point Likert scale. The resilience scale Korean version, which was verified by Lee, Lee, and Choi (2017) was used in this study. The Cronbach’s alpha in the original study was .92, and that in this study was .94.

#### QOL

The KIDSCREEN 52 health questionnaire for children and young people developed by the KIDSCREEN group was used to evaluate and compare the QOL between adolescent blood cancer survivors and adolescents with CHD [24]. This instrument consisted of 52 items and 10 subscales of physical activities and health (5 items), feelings (6 items), general mood (7 items), self-perception (5 items), free time (5 items), family and home life (6 items), money matters (3 items), friends (6 items), school and learning (6 items), and bullying (3 items). KIDSCREEN 52 is performed using a 5-point Likert self-report scale. A higher score indicates higher QOL. The KIDSCREEN Korean version, which was verified by Hong [25], was used to test the Korean adolescents with chronic illness. The Cronbach’s alphas in the previous study ranged from .77 to .95 for verifying the reliability of the KIDSCREEN Korean version [25], and the Cronbach’s alphas in this study were from .75 to .95.

#### General characteristics

General characteristics were collected by self-report questionnaire that consisted of gender, age, school level, religion, parent marital status and education level, and perceived economic status. Clinical diagnosis was assessed through the medical record. The diagnosis for adolescents blood cancer was classified as acute lymphoblastic leukemia, acute myeloid leukemia, lymphoma, and myeloma. The diagnosis for CHD was classified as simple, moderate severity, and great complexity, which was the classification of the Task Force 1 of the 32nd Bethesda Conference of the American College of Cardiology [26].

### Data analysis

SPSS 20.0 was used to analyze the data. The descriptive analysis was conducted to evaluate the homogeneity of two groups: adolescent blood cancer survivors and adolescents with CHD. T-test was used to compare the resilience and QOL between adolescent blood cancer survivors and adolescents with CHD. ANCOVA was conducted to evaluate the resilience and QOL according to group (adolescent blood cancer survivors vs. adolescents with CHD) and school level. The absolute values of skewness and excess kurtosis were calculated to be lower than one, which were in acceptable ranges for assuming a normal distribution [27]. School level was included as an independent variable because it was significantly different between the groups in the previous analysis.

## Results

### Diagnoses of the two groups

As shown in Table 1, Fifty-eight (53.2%), fourteen (14.9%), and twenty-two (23.4%) participants in the group of adolescent blood cancer survivors had been diagnosed leukemia, lymphoma, and myeloma, respectively. Fifteen (18.5%), thirty-three (40.7%), and thirty-three (40.7%) adolescents with CHD had been diagnosed with ‘simple’, ‘moderate severity’ and ‘great complexity’, respectively.

**Table 1:** Diagnoses of the participants

Adolescent blood cancer survivors		Adolescents with CHD	
Diagnosis	n (%)	Diagnosis	n (%)
Acute lymphoblastic leukemia	50 (53.2)	Simple	15 (18.5)
Acute myeloid leukemia	8 (8.5)	Moderate severity	33 (40.7)
Lymphoma	14 (14.9)	Great complexity	33 (40.7)
Myeloma	22 (23.4)		
Total	94		81

### Homogeneity of the two groups

s shown in Table 2, general characteristics were not significantly different between adolescent blood cancer survivors and adolescents with CHD, with the exception of school level (*p* = .02). Seventy-five adolescent blood cancer survivors (79.8%) attended middle or high school, and 11 participants (11.7%) attended college, while 52 adolescents with CHD (64.2%) attended middle or high school, and 24 participants (29.6%) attended college. Sixty-two (66.0%) and 49 (60.5%) adolescent blood cancer survivors and adolescents with CHD, respectively, were male. Forty-two (45.2%) and 34 (42%) adolescent blood cancer survivors and adolescents with CHD, respectively, were religious. Eighty-two adolescent blood cancer survivors (89.1%) perceived their economic status as above average, whereas 71 adolescents with CHD (87.6%) perceived their economic status as above average.

**Table 2:** General characteristics of the participants and homogeneity of the two groups

Variables		Adolescent blood cancer survivors	Adolescents with CHD	X^2^/t (p)
Gender	Male	62 (66.0)	49 (60.5)	0.56 (.53)
	Female	32 (34.0)	32 (39.5)	
Age in years		16.27 ± 2.04	16.48 ± 2.33	−0.65 (.52)
School	Middle	34 (36.2)	29 (35.8)	10.07 (.02)
	High	41 (43.6)	23 (28.4)	
	College	11 (11.7)	24 (29.6)	
	Others	8 (8.5)	5 (6.2)	
Siblings	No	16 (17.0)	15 (18.5)	0.07 (.48)
	Yes	78 (83.0)	66 (81.5)	
Religion	No	51 (54.8)	47 (58)	0.18 (.76)
	Yes	42 (45.2)	34 (42)	
Parental marital status	Married	83 (88.3)	72 (88.9)	0.02 (.55)
	Divorce or bereaved	11 (11.7)	9 (11.1)	
Father’s education level	<High school	3 (3.3)	4 (5.7)	3.57 (.17)
	High school	42 (45.7)	22 (31.4)	
	College or beyond	47 (51.1)	44 (62.9)	
Mother’s education level	<High school	1 (1.1)	2 (2.7)	1.03 (.59)
	High school	43 (46.7)	30 (41.1)	
	College or beyond	48 (52.2)	41 (56.2)	
Perceived economic status	Bad	10 (10.9)	10 (12.3)	2.81(.25)
	Average	61 (66.3)	44 (54.3)	
	Good	21 (22.8)	27 (33.3)	

Others: leave of absence from school or graduated

### Comparison of resilience and QOL between adolescent blood cancer survivors and adolescents with CHD

The resilience of adolescent blood cancer survivors was significantly lower than that of adolescents with CHD (*p* = .03). The resilience mean scores were 121.83 and 129.27 in adolescent blood cancer survivors and adolescents with CHD, respectively. The resilience mean score of adolescent blood cancer survivors was low according to the criteria by Wagnild.^16^ With regard to QOL, the mean score of adolescent blood cancer survivors was 202.99, and that of adolescents with CHD was 202.14; therefore, there was not a significant difference between groups (*p* = .75) (Table 3).

**Table 3:** Comparison of resilience and QOL in adolescent blood cancer survivors and adolescents with CHD

Variable	Adolescent blood cancer survivors	Adolescents with CHD	T (p)
Resilience	121.83 ± 27.19	129.27 ± 17.19	−2.19 (.03)
Personal Competence	84.87 ± 19.71	90.19 ± 13.14	−2.12 (.04)
Acceptance of self and life	36.96 ± 8.44	39.09 ± 5.49	−2.01 (.04)
QOL	202.99 ± 27.30	202.14 ± 25.41	0.21 (.83)

### Resilience and QOL according to group and school level

**Fig. 1:**
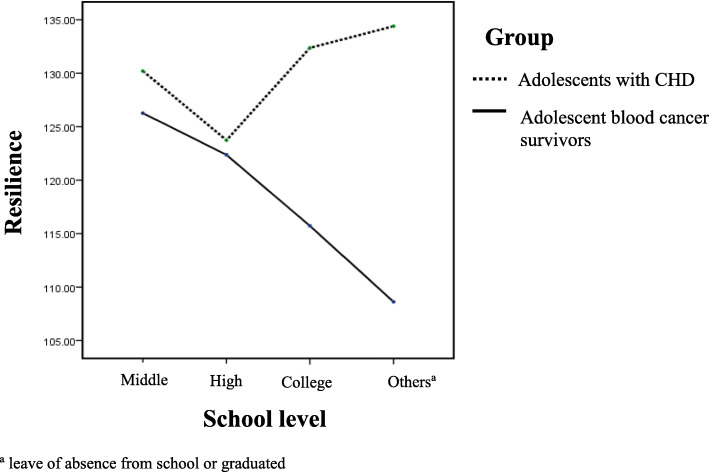
Resilience according to group and school level. ^a^ leave of absence from school or graduated

Table 4 and Fig. 1 showed the results of ANCOVA. Because school level was significantly different in the previous analysis for the homogeneity of the two groups, we assigned group and school level as independent variables. The resilience of adolescent blood cancer survivors was significantly lower than that of adolescents with CHD (*p* = .01). The resilience did not differ significantly according to school level (*p* = .58); however, adolescent blood cancer survivors had a significantly lower resilience than adolescents with CHD in the participants in the category of others, including those who had taken a leave of absence from school or graduated. The QOL of adolescent blood cancer survivors was not different from that of adolescents with CHD (*p* = .41) (Table 4).

**Table 4:** The factors for resilience and QOL analyzed by ANCOVA

Dependent Variable	Independent variables	df	F	p
Resilience	Model	7	1.61	.14
	Group by diagnosis	1	7.27	.01
	School	3	0.44	.58
	Group x School	3	1.51	.22
QOL	Model	7	1.12	.35
	Group by diagnosis	1	0.67	.41
	School	3	2.35	.07
	Group x School	3	0.58	.63

## Discussion

Our findings indicate that the resilience of adolescent cancer survivors was significantly lower than that of adolescents with CHD. Adolescents blood cancer and CHD were perceived as chronic illnesses, indicating that adolescent blood cancer survivors and adolescents with CHD live with uncertainty [28], similar to other adolescents with chronic illness [29]. adolescents with CHD experienced uncertainty due to repeated treatment and complications [30], while adolescent cancer survivors experienced uncertainty due to the possibility of relapse and the side effects of treatment [31]. The repeated treatment for adolescents with CHD was planned out as far as possible, and the education program focused on giving adolescents with CHD information for how to prevent complications [32]. However, the relapse possibility cannot be determined by education; therefore, adolescent blood cancer survivors might experience more uncertainty than adolescents with CHD. It is difficult for adolescent blood cancer survivors to perceive illness controllability and self-regulation, because there are no known methods to prevent second cancer or relapse. The parallel model which was developed by Leventhal stated that health threats generate both fear control and danger control [33]. Fear control and danger control referred to the parallel actions that are undertaken for their efficacy in reducing the negative emotions and the threat themselves [33]. The information about health threats lead to the perception of fear and danger, then, action plans are undertaken to reduce them [33]. The parallel process model proposed that cognition of health threats was a necessary condition for danger control [33]. However, adolescent blood cancer survivors who recognized their health threat could not control the danger of second cancer or relapse. Illness controllability was associated with good psychological and physical adjustment [29]. Self-regulation involves adherence to daily tasks such as nutrition and prevention of infection and improvement of health outcomes [12]. However, adolescent blood cancer survivors had difficulties perceiving illness controllability and self-regulation. High illness controllability and self-regulation were associated with high resilience and high frequency of problem solving [34]. The adolescent blood cancer survivors in this study seemed to show lower resilience than adolescents with CHD because they had difficulty achieving illness controllability and self-regulation.

In addition, adolescent blood cancer survivors endure longer duration of treatment compared to adolescents with CHD. For example, children and adolescents with acute lymphoblastic leukemia receive several months or years of treatment [35]. Many blood cancer survivors experience difficulties at school [12] and grade retention [36] due to the long treatment period, requiring absence from school. Peer relationships and participation at school facilitate disease adaptation and improvement of resilience [37]. Our finding also showed a large difference in resilience between adolescent blood cancer survivors and adolescents with CHD for participants who had required a leave of absence from school or graduated. Difficult situations such as longer duration of treatment and grade retention could be associated with lower resilience of adolescent cancer survivors in comparison to adolescents with CHD. The psychological factors associated with resilience were positive emotion, acceptance, spirituality, social support, and active coping style [38]. Therefore, health-providers need to develop a resilience improvement program focused on positive emotion, acceptance, and social support in addition to active coping styles such as education for adolescents blood cancer survivors.

The QOL of adolescent cancer survivors was not significantly different from that of adolescents with CHD. A systematic review study reported that the studies about the QOL of adolescents with chronic disease were heterogeneous; the majority of the studies reported a significantly higher risk of impairment on QOL, whereas other studies reported a significantly lower risk of impairment on QOL in adolescents with chronic disease [39]. Hamner et al. [40] used hierarchical regression analysis in their study of adolescents cancer patients and demonstrated that QOL was more highly associated with parental chronic stress than with time since diagnosis. Therefore, the QOL of adolescents with chronic disease could be more highly affected by individual psychological factors, protective factors, and the adaptation process than with the medical diagnosis. Future research is needed to investigate the variables related to QOL of adolescents blood cancer survivors.

Our study has several limitations. Firstly, resilience and QOL which are the dependent variables in the present study were influenced by others factors such as severity of illness and comorbidity. However, adolescent who can lead a daily life were included and adolescent who were currently suffering from complications were excluded in this study. And adolescent cancer survivors and adolescents with CHD were recruited from different hospitals in the same city. Therefore, further research need to be carried out in view of these exogenous variables. Secondly, the present study was a cross-sectional study and the time after diagnosis and the time after treatment were not included as variables. The time after diagnosis and the time after treatment can be important variables which influence on resilience and QOL. Further longitudinal studies considering time after diagnosis and treatment are needed. Thirdly, the mean age of this study sample was 16 years and the subjects were an older paediatric group. Future research for exploring resilience and QOL of younger age groups and for investigating the effect of age on resilience and QOL are needed.

## Conclusion

This study showed that the resilience of adolescent cancer survivors was significantly lower than that of adolescents with CHD. Adolescent cancer survivors experienced uncertainty due to relapse possibility and side effects of treatment; however, relapse possibility cannot be determined by education. It is difficult for adolescent cancer survivors to perceive illness controllability and self-regulation. In addition, living in a difficult situation such as longer duration of treatment or grade retention could be factors associated with lower resilience of adolescent cancer survivors compared to adolescents with CHD. Due to advanced medical treatments, adolescents blood cancer as well as CHD are regarded as chronic disease. However, the experiences of adolescent blood cancer survivors were different from those of adolescents with CHD even though they are of the same ages. Adolescents with chronic disease have a different level of illness controllability and self-regulation according to their disease and situation. Therefore, health-providers need to develop the specific programs for improving resilience and QOL of adolescents with chronic illness with focusing their characteristics and situations.

## Data Availability

The datasets generated and/or analysed during the current study are available from the corresponding author on reasonable request.

## Abbreviations

QOL: Quality of Life
CHD: Congenital Heart Disease
ARM: Adolescent Resilience Model
